# Tuina Inhibits Synaptic Plasticity Through the Astrocytic NDRG2/GLT‐1 Pathway to Alleviate Neuropathic Pain

**DOI:** 10.1111/jcmm.71013

**Published:** 2026-01-07

**Authors:** Huanzhen Zhang, Lechun Chen, Jingjing Jiang, Limei Huang, Hongye Huang, Lanting Huang, Shuijin Chen, Zhigang Lin

**Affiliations:** ^1^ Rehabilitation Hospital Affiliated to Fujian University of Traditional Chinese Medicine Fuzhou China; ^2^ Fujian Key Laboratory of Rehabilitation Technology Fuzhou China; ^3^ Fujian University of Traditional Chinese Medicine Fuzhou China

**Keywords:** astrocytes, NDRG2/GLT‐1, neuropathic pain, synaptic plasticity, Tuina

## Abstract

While Tuina has demonstrated clinical efficacy in alleviating neuropathic pain (NP) induced by peripheral nerve injury, the underlying molecular mechanisms remain unclear. Synaptic plasticity in the spinal dorsal horn (SDH) is a key regulator of pain processing, wherein astrocytes play a central role. Regulation of N‐myc downstream‐regulated gene 2 (NDRG2) and glutamate transporter 1 (GLT‐1) is particularly important in NP modulation. Accumulating evidence suggests that Tuina exerts analgesic effects by inhibiting aberrant synaptic plasticity. This study explored whether Tuina regulates synaptic plasticity through astrocytes to attenuate NP. Using an in vivo model, Tuina or the astrocytic inhibitor fluorocitrate was administered to a chronic constriction injury (CCI)‐induced NP rat model for 14 days. Tuina significantly alleviated pain hypersensitivity in CCI rats, improved structural damage, suppressed astrocytic activation, reduced glutamate accumulation and restored the SDH's expression of proteins linked to synaptic plasticity. These effects were associated with inhibition of astrocytic NDRG2 and upregulation of GLT‐1. Conversely, NDRG2 overexpression through AAV impaired Tuina's ability to promote glutamate transport, leading to glutamate accumulation, enhanced excitatory transmission and reduced analgesic efficacy. In conclusion, Tuina alleviates NP by modulating the astrocytic NDRG2/GLT‐1 pathway, reducing synaptic glutamate levels and normalising synaptic plasticity. These findings provide new mechanistic insights into Tuina's analgesic action and have identified NDRG2/GLT‐1 as a possible therapeutic target for NP treatment.

## 1 Introduction

Neuropathic pain (NP), which usually presents as a chronic, recurrent and refractory illness, is characterised by spontaneous pain, hyperalgesia, and allodynia [1]. Epidemiological data suggest that NP affects 7%–10% of the population and constitutes 20%–25% of chronic pain cases. Its high prevalence, coupled with limited treatment efficacy, presents a major public health challenge [2]. First‐line pharmacological options, such as gabapentin and tricyclic antidepressants, often provide only partial relief and are limited by adverse central nervous system effects. This has spurred the need for safer and more effective alternatives. Among these, physical therapies such as thermotherapy for analgesia and relaxation, electromagnetic therapies for neuromodulation and photobiomodulation for tissue repair, alongside mechanical interventions such as traction, have been integrated into clinical practice with demonstrated benefits for some patients [3]. Nonetheless, their effectiveness varies substantially across individuals, which restricts broad applicability. Therefore, developing more comprehensive and multidimensional approaches to NP management remains crucial.

Within this paradigm, Tuina therapy, a distinctive manual intervention in traditional Chinese medicine, has shown unique benefits in NP management [4]. A recently conducted multicenter survey in China reported that 66.8% of chronic pain patients preferred Tuina as their primary treatment, citing both its clinical effectiveness and broad social acceptance. Importantly, Tuina is associated with a markedly lower incidence of adverse events than pharmacological approaches [5]. Despite these promising outcomes, the mechanisms underlying Tuina‐induced analgesia remain poorly defined.

Spinal dorsal horn (SDH) is a critical region for pain transmission and integration, and maladaptive synaptic plasticity within this region is a major contributor to NP [6]. Peripheral nerve damage causes the SDH to undergo long‐term potentiation (LTP), leading to abnormal amplification of nociceptive signalling [7]. Our prior work demonstrated that Tuina markedly suppressed SDH LTP in rats with chronic constriction injury (CCI) and ameliorated synaptic ultrastructural damage, suggesting that Tuina modulates SDH synaptic plasticity to produce analgesic benefits [8, 9].

Emerging evidence highlights astrocytes as a central regulator of synaptic plasticity. By dynamically ensheathing synapses and maintaining neurotransmitter homeostasis, astrocytes exert profound control over neuronal signalling [10]. In particular, the astrocytic N‐myc downstream‐regulated gene 2 (NDRG2) regulates the expression of glutamate transporter 1 (GLT‐1), which clears glutamate from the synaptic cleft and reverses maladaptive plasticity, thereby representing a promising therapeutic target in NP [11].

Although prior research indicating that Tuina can inhibit astrocyte activation and mitigate NP [12], its role in the astrocytic regulation of synaptic plasticity remains unclear. We hypothesized that Tuina alleviates NP by modulating the astrocyte NDRG2/GLT‐1 pathway, thereby enhancing glutamate uptake and suppressing pathological plasticity in the SDH. To test this, we established a CCI rat model to examine astrocytic NDRG2/GLT‐1 signalling following Tuina treatment, alongside ultrastructural and biochemical changes in astrocytes and synapses. This approach aimed to clarify the mechanistic role of the NDRG2/GLT‐1 pathway in Tuina's analgesic effects, offering new insights into its therapeutic potential.

## 2 Materials and Methods

### 2.1 Experimental Animals

Healthy male Sprague–Dawley (SD) rats weighing 180–220 g at 8 weeks of age were used in the experiment. They were acquired from Shanghai SLAC Laboratory Animal Co. Ltd. (licence number: SYXK [Hu] 2020–0009). The rats lived in regulated environments with free access to food and water and a 12‐h light/dark cycle with a temperature range of 22°C–25°C and 50% humidity. To minimise stress during pain‐related behavioural tests, rats were handled daily to acclimate them to experimenters. All procedures were authorised by the Fujian University of Traditional Chinese Medicine's Animal Ethics Committee (FJTCM IACUC 2023113) and conducted in accordance with relevant animal welfare guidelines.

### 2.2 Model Establishment

The CCI rat model of NP was established to replicate progressive nerve injury according to the method of Bennett and Xie [13]. Briefly, rats were sedated with intraperitoneal administration of 4% sodium pentobarbital (0.2 mL·100 g^−1^) and placed laterally. After shaving and disinfecting the thigh, a longitudinal incision was made along the femur and blunt dissection was performed to expose the ipsilateral sciatic nerve. Using four 4–0 absorbable sutures, the nerve was loosely tied 1 mm from the trifurcation [14]. Following irrigation with sterile saline, the nerve was repositioned and the wound closed in layers. Penicillin sodium powder was applied to prevent infection, and the rats were warmed postoperatively before being returned to cages with ad libitum food and water.

### 2.3 Tuina Intervention

Tuina intervention was performed according to the kneading method, previously shown to alleviate NP [15]. The rats were habituated to the Tuina restrainer for 30 min daily for 3 days before treatment. Each session included a 10‐min adaptation period, followed by kneading of the Weizhong acupoint (BL40) on the operative side using a self‐developed smart thumb sleeve (patent number: ZL201821318407.9) designed for rat Tuina. The device had a circular massage probe (0.5 cm in diameter) that minimised the contact area for a better fit on the rats. It also had a micro‐pressure sensor, with kneading parameters acquired from a wrist‐worn control board and wirelessly transmitted to a smartphone for real‐time assessment. Based on prior studies, force was set at 4–6 N, frequency at 120 cycles/min and duration at 10 min/session, administered once daily for 14 days [10]. After each session, the rats rested individually for 1 min to prevent social interference and ensure intervention effectiveness.

### 2.4 Drug Administration

#### 2.4.1 Administration of Fluorocitrate

FCA (Sigma, USA), an astrocyte inhibitor, was dissolved in 1 N hydrochloric acid and neutralised with Na₂SO₄ and PBS. The supernatant was collected, diluted with 4.8 mL of saline, and adjusted to 1 nmol/μL following 5 min of centrifugation at 12,000 rpm [16]. FCA was intrathecally administered into the L5–6 intervertebral space at 10 μL/day for 14 days.

#### 2.4.2 Administration of Adeno‐Associated Virus

The NDRG2 overexpression virus (rAAV‐GfaABC1D‐NDRG2‐P2A‐mCherry‐WPRE‐SV40 pA) and the control vector (rAAV‐GfaABC1D‐mCherry‐WPRE‐SV40 pA) were purchased from Wuhan Shumi Brain Science and Technology Co. Ltd. A total of 20 μL of viral suspension (titre: 2.00E+12 vg/mL) was injected vertically into the L5–6 spinous process gap 21 days before CCI induction. Safe and appropriate injection was verified by tail‐flick reflex.

### 2.5 Experimental Design

**FIGURE 1:**
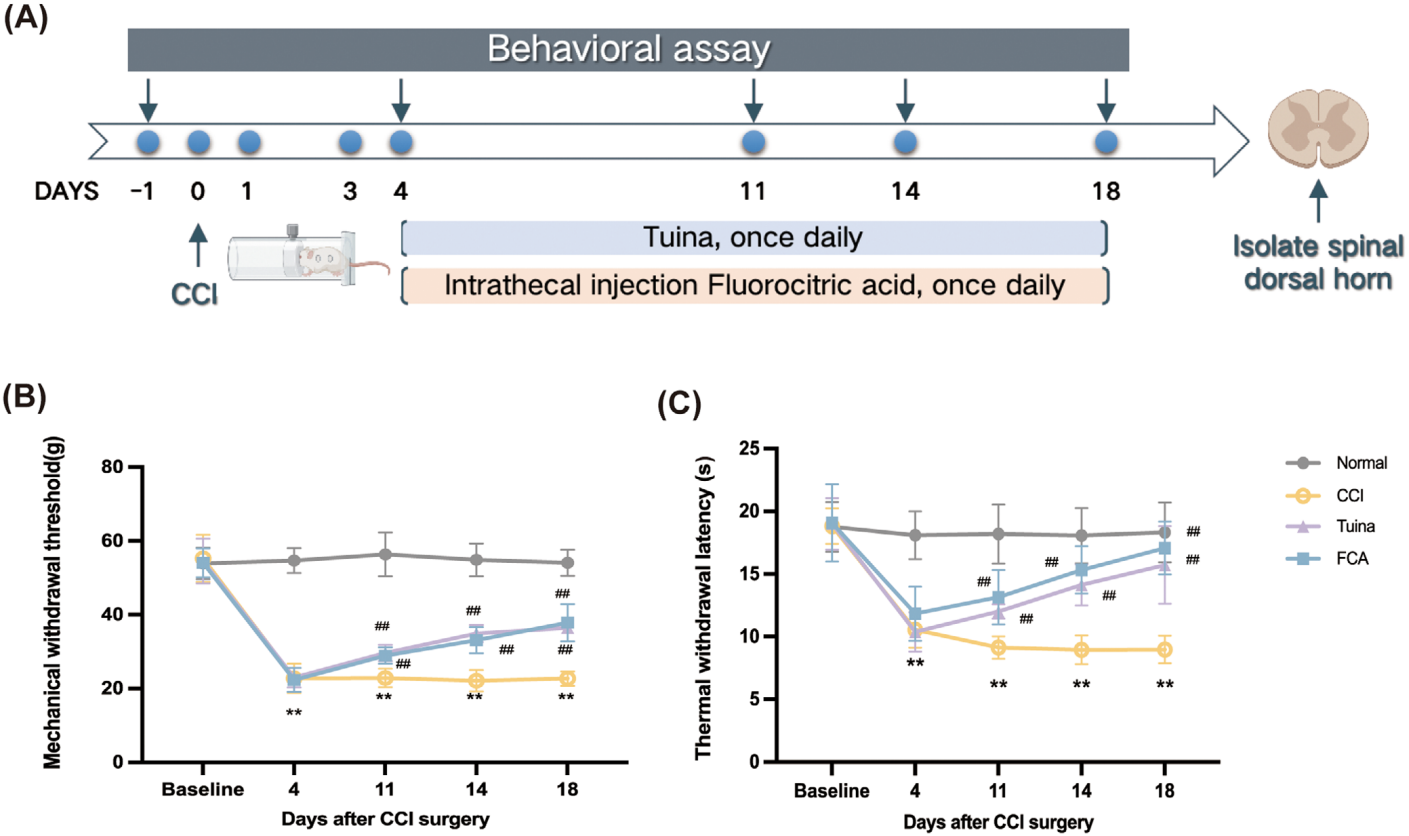
Tuina improved CCI‐induced pain hypersensitivity. (A) Study protocol for Experiment 1. (B) Changes in MWT among different groups of rats. (C) Changes in TWL among different groups of rats. *n* = 12. **p < 0.01. versus normal group. ^##^ *p* < 0.01. versus CCI group.

Experiment 1 was designed to evaluate the role of Tuina in regulating the astrocyte NDRG2/GLT‐1 pathway in NP; the rats were randomly assigned to four groups (*n* = 18/group): normal, CCI, Tuina, and FCA. On day 4 after CCI, the Tuina group received kneading treatment; the FCA group received intrathecal FCA injections; the normal group received only routine handling, and the CCI group underwent CCI without any further intervention. Pain behaviours were assessed at defined time points, after which the rats were euthanized and spinal cord tissues were harvested for further analysis (Figure 1A).

Experiment 2 was designed to induce NDRG2 overexpression through intrathecal AAV delivery, which confirmed that the astrocyte NDRG2/GLT‐1 pathway is a critical mediator of Tuina‐induced synaptic plasticity in NP. To validate viral effectiveness, the rats were initially randomised into two groups: AAV NDRG2 and vector control (*n* = 8 each). Subsequently, the rats were further assigned to four experimental groups: CCI + Vector, CCI + AAV NDRG2, CCI + Vector + Tuina, and CCI + AAV NDRG2 + Tuina (*n* = 15 per group). NDRG2 expression, temporal changes in pain behaviour and spinal cord tissue samples were assessed for subsequent analyses (Figure 4A).

### 2.6 Behavioural Tests

#### 2.6.1 Mechanical Withdrawal Threshold (MWT)

Mechanical allodynia was evaluated using an electronic Von Frey apparatus. Lower threshold values indicated greater pain sensitivity. Before testing, the rats were acclimated for 30 min in transparent acrylic chambers elevated 40 cm above a metal mesh platform. In the calm state, a calibrated probe was placed perpendicularly to the plantar surface of one hind paw until a withdrawal or licking response occurred. MWT was calculated using the average of the five trials that each rat underwent at 5‐min intervals; the highest and lowest values were eliminated.

#### 2.6.2 Thermal Withdrawal Latency (TWL)

Thermal nociception was assessed using a plantar thermal stimulator. The rats were placed in transparent chambers on a glass plate and acclimated for 30 min. The light source, set at 50% intensity with a 30‐s cutoff, was directed onto the hind paw and paw withdrawal latency was automatically recorded. Each animal underwent five trials with 5‐min intervals, and the average of the middle three values was taken as the TWL.

### 2.7 Transmission Electron Microscopy (TEM)

The ultrastructure of astrocytes and their association with synapses in the L4–L6 SDH were examined using TEM. Tissue blocks (1 mm^3^) were fixed with glutaraldehyde and osmium tetroxide. Uranyl acetate and lead citrate were used to stain ultrathin slices (50–100 nm). Astrocytes were distinguished by their stellate shape, transparent cytoplasm, and glycogen granules; synapses were recognised by intact pre‐ and postsynaptic specialisations. Quantitative measures included astrocyte–synapse contact area and perisynaptic astrocytic process (PAP) tip length, analysed using ImageJ software.

### 2.8 Immunofluorescence (IF) Staining and Co‐Staining

Astrocytic expression of glial fibrillary acidic protein (GFAP) and co‐localization of NDRG2 and GLT‐1 were assessed by IF. Glutamatergic synapses were visualised by co‐staining of vesicular glutamate transporter 1 (VGLUT1) and postsynaptic density protein 95 (PSD95). Primary antibodies against NDRG2 (Abcam; ab174850, 1:100), GLT‐1 (Proteintech; 22,515–1‐AP, 1:100), GFAP (Abcam; ab7260, 1:5000), VGLUT1 (CST, 47181S, 1:200) and PSD95 (CST, 36233S, 1:100) were added to deparaffinised and blocked L4‐L6 SDH tissue sections. The sections were incubated overnight at 4°C. A fluorescent microscope was used to image the tissue slices after secondary antibody labelling.

### 2.9 Glutamate and *γ*‐Aminobutyric Acid Concentration Assay

Glutamate and γ‐aminobutyric acid (GABA) levels in L4‐L6 SDH tissues were quantified using commercial kits (Wuhan Yunkelong Biotechnology Co. Ltd., CES122Ge for glutamate; CEA900Ge for GABA). Absorbance was quantified at 450 nm and concentrations were determined in accordance with manufacturer instructions.

### 2.10 Real‐Time qPCR Assay

The mRNA expression of NDRG2 and GLT‐1 in the L4‐L6 SDH tissues was quantified using RT‐qPCR assay. Total RNA was isolated using TRIzol, reverse‐transcribed and amplified with GAPDH serving as the internal reference. We used the 2^−△△CT^ technique to calculate the relative expression. Table 1 shows the primer sequences used in this study.

**TABLE 1:** Primer sequences of target genes.

Primer name	Primer sequence(5′‐3′)	Amplicon length
Rat NDRG2 F‐primer	CTCATGAAGATGCCGTGGTG	61 bp
Rat NDRG2 R‐primer	AGGAATGAGGTCTGTGTGGG	
Rat GLT‐1 F‐primer	CCGAGGGTGCCAACAATATG	139 bp
Rat GLT‐1 R‐primer	GTCAGTGAGAGCAGGAGGTT	
Rat GAPDH Forward	GGCAAGTTCAACGGCACAGT	128 bp
Rat GAPDH Reverse	ATGACATACTCAGCACCGGC	

### 2.11 Western Blot

L4‐L6 SDH tissues were lysed for protein analysis, and proteins were quantified using the bicinchoninic acid assay. The samples were subjected to electrophoresis by denaturing the samples and transferring them onto a polyvinylidene difluoride membrane, after which they were blocked with 5% nonfat milk. Primary antibodies against Syp (Proteintech; 17,785–1‐AP, 1:5000), Syt‐1 (Proteintech; 14,511–1‐AP, 1:4000), NDRG2 (Abcam; ab174850, 1:1000), GLT‐1 (Proteintech; 22,515–1‐AP, 1:5000), ATP1A1 (Proteintech; 14,418–1‐AP, 1:5000), ATP1B1 (Proteintech; 15,192–1‐AP, 1:1000), ACTIN (Servicebio; GB15001, 1:5000) and GAPDH (CST, D16H11, 1:1000) were used to probe the membranes. The samples were then incubated overnight at 4°C. Following washing, the membranes were incubated with secondary antibodies and ImageJ software was used for analysis.

### 2.12 Statistical Analysis

SPSS 26.0 was used for data analysis. Data of normally distributed variables were expressed as mean ± SD. Repeated‐measures analysis of variance (ANOVA) was used to compare data within groups across time points and one‐way ANOVA was used to assess differences between groups. Post hoc testing was conducted using LSD‐t or Games‐Howell, as appropriate. A *p*‐value of < 0.05 was considered statistically significant.

## 3 Results

### 3.1 Tuina Improves **CCI** ‐Induced Pain Hypersensitivity

To evaluate the therapeutic effects of Tuina on NP, a CCI rat model was established (Figure 1A). Pain behaviour assessments demonstrated that, compared with the normal group, both the MWT and TWL were significantly reduced in the CCI, Tuina, and FCA groups, confirming the induction of mechanical and thermal hypersensitivity. From days 7 to 14 of Tuina intervention, MWT significantly increased and TWL was markedly prolonged relative to the CCI group. A comparable improvement was observed in the FCA group following intrathecal astrocyte inhibition during the same period. No significant differences were detected between the Tuina and FCA groups (Figure 1B,C). These findings indicate that Tuina markedly alleviates pain hypersensitivity in the CCI model, with an efficacy similar to that of astrocyte inhibition.

### 3.2 Tuina Improves **CCI** ‐Induced Activation of Astrocytes, Glutamate Accumulation and Synaptic Remodelling in the **SDH**

**FIGURE 2:**
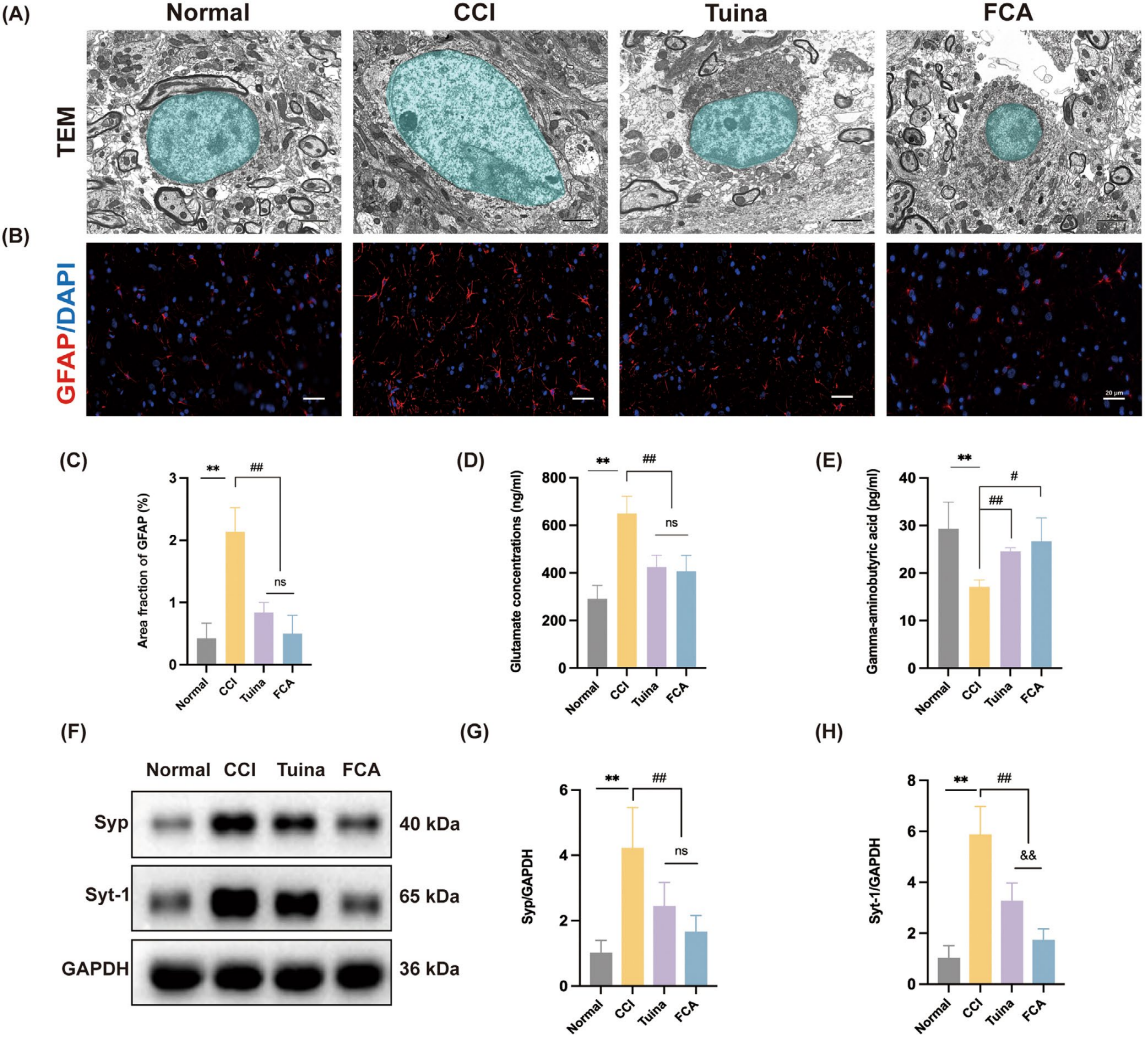
Tuina improved CCI‐induced activation of astrocytes, glutamate accumulation and synaptic remodelling in SDH. (A) TEM images showed the ultrastructural morphology of astrocytes in each group (astrocytes are pseudo‐coloured in blue). Scale bar, 2 μm. (B) IF images showed GFAP expression in SDH across different groups. Scale bar, 50 μm. (C) Quantitative analysis of GFAP expression in each group. *n* = 4. (D, E) Concentrations of glutamate and *γ*‐aminobutyric acid in each group. *n* = 6. (F) Protein expression levels of Syp and Syt‐1 in SDH. GAPDH was used as the internal control. (G, H) Relative protein expression levels of Syp (G) and Syt‐1 (H) in each group. *n* = 5. Data are presented as mean ± SD. **p < 0.01. versus normal group. ^#^ *p* < 0.05, ^##^ *p* < 0.01. versus CCI group. ^&&^ *p* < 0.01. versus Tuina group.

To further confirm the effects of Tuina on astrocytes, neurotransmitters and synaptic regulation, multiple indices were assessed. TEM revealed that astrocytes in the normal group exhibited moderate volume, well‐organised structure, intact membranes, evenly distributed cytoplasm and morphologically normal organelles, including mitochondria, endoplasmic reticulum and Golgi apparatus. By contrast, astrocytes in the CCI group displayed pronounced swelling, condensed nuclear staining, ruptured membranes, increased cytoplasmic density, damaged mitochondria with disrupted cristae and perilesional lysosomes. In the Tuina group, astrocytic alterations were attenuated, with only mild swelling, uniform nuclear staining, relatively intact membranes, loosely distributed cytoplasm and minimal mitochondrial damage. The FCA group showed a similar degree of protection (Figure 2A).

IF analysis of GFAP expression revealed markedly elevated fluorescence intensity in the CCI group, accompanied by enlarged astrocytic somata and elongated, proliferative processes. By contrast, GFAP intensity was significantly reduced in both the Tuina and FCA groups, which also exhibited normalisation of astrocytic morphology (Figure 2B,C). These data suggest that Tuina effectively suppresses astrocyte activation.

Neurotransmitter profiling using ELISA demonstrated that CCI markedly elevated glutamate and reduced GABA concentrations in the SDH. Tuina intervention or FCA administration significantly reversed these changes, lowering glutamate and restoring GABA levels. No significant differences were observed between the two treatments (Figure 2D,E), suggesting that Tuina mitigates CCI‐induced excitatory–inhibitory imbalance by limiting glutamate accumulation.

Western blot analysis further examined synaptic plasticity proteins. Expression of synaptophysin (Syp) and synaptotagmin‐1 (Syt‐1) was significantly increased in the CCI group. Both Tuina and FCA treatments downregulated these proteins. While the reduction in Syp was comparable between the two groups, Syt‐1 suppression was more pronounced with FCA (Figure 2F–H). Collectively, these findings indicate that Tuina suppresses astrocyte activation, reduces glutamate accumulation, and modulates synaptic remodelling.

### 3.3 Tuina Inhibits **NDRG2** Expression and Enhances **GLT** ‐1 Expression in the **SDH** of **CCI** Rats

**FIGURE 3:**
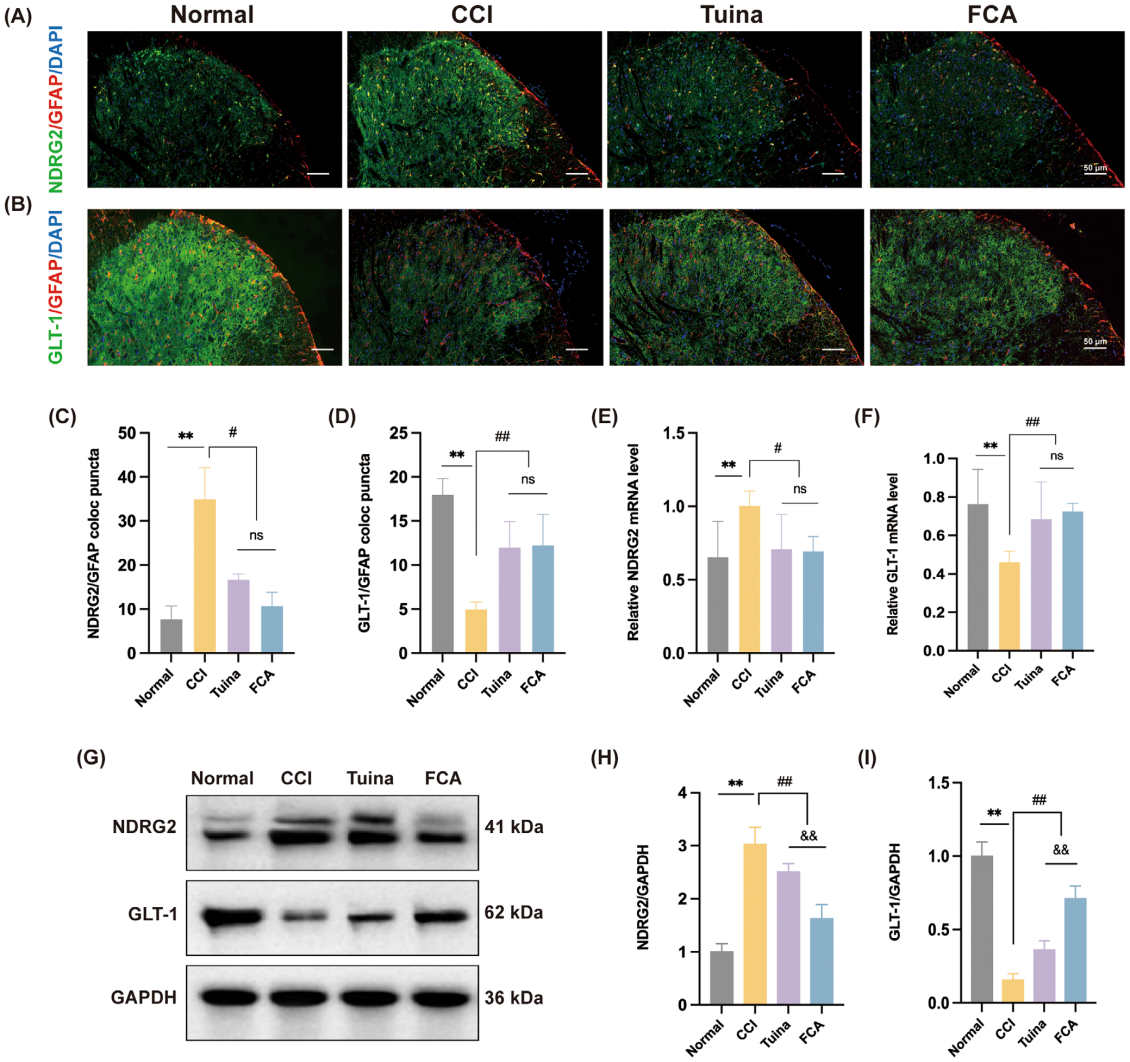
Tuina inhibited NDRG2 expression and enhanced GLT‐1 expression in the SDH of CCI rats. (A) Representative immunofluorescence images of SDH nuclei stained with NDRG2 (green), GFAP (red) and DAPI (blue) among the groups. Scale bar, 50 μm. (B) Representative immunofluorescent images of SDH nuclei stained with GLT‐1 (green), GFAP (red) and DAPI (blue) among groups. Scale bar, 50 μm. (C, D) Quantification analysis of the number of NDRG2/GFAP (C) and GLT‐1/GFAP (D). *n* = 4. (E, F) Expression levels of NDRG2 and GLT‐1 mRNA among different groups. *n* = 5. (G) SDH NDRG2 and GLT‐1 protein levels. GAPDH was used as the internal control. (H‐I) Relative protein expression levels of NDRG2 (H) and GLT‐1 (I) in each group. *n* = 5. Data are presented as mean ± SD. **p < 0.01 versus normal group. ^#^ *p* < 0.05, ^##^ *p* < 0.01 versus CCI group. ^&&^ *p* < 0.01 versus Tuina group.

NDRG2 plays a pivotal role in regulating synaptic glutamate homeostasis. Under NP conditions, NDRG2 translocates to the nucleus and cell membrane, represses GLT‐1 transcription, decreases glutamate uptake, enhances synaptic transmission and contributes to hyperalgesia. To investigate Tuina's effect on NDRG2/GLT‐1 signalling, tissue co‐staining was performed. Both proteins co‐localised with GFAP, confirming astrocytic expression (Figure 3A,B). In the CCI group, NDRG2/GFAP double‐positive cells were significantly increased, whereas GLT‐1/GFAP double‐positive cells were reduced. Tuina and FCA treatments reversed these alterations, decreasing NDRG2/GFAP and increasing GLT‐1/GFAP co‐localization. No significant differences were observed between the two treatments (Figure 3C,D). Western blot and RT‐qPCR assays corroborated these findings (Figure 3E–I). These results suggest that Tuina downregulates astrocytic NDRG2 while enhancing GLT‐1 expression, thereby promoting glutamate uptake and attenuating aberrant synaptic plasticity.

### 3.4 AAV‐NDRG2 Weakens the Analgesic Effect of Tuina in **CCI** Rats

**FIGURE 4:**
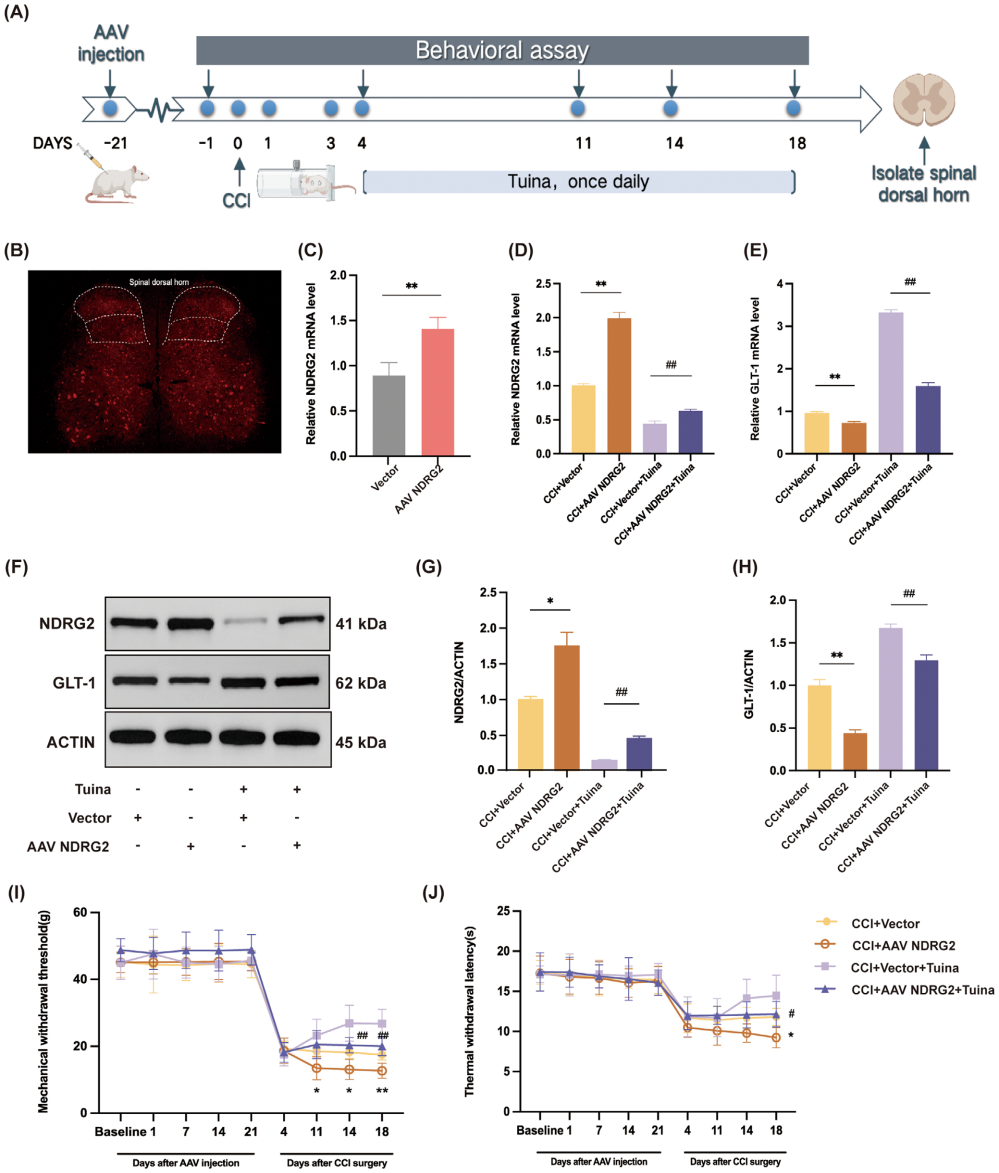
AAV‐NDRG2 weakens the analgesic effect of Tuina in CCI rats. (A) Study protocol for Experiment 2. (B) Representative diagram of AAV‐NDRG2 infection in SDH. Scale bar, 400 μm. (C) NDRG2 expression via RT‐qPCR assay. *n* = 5. **p < 0.01. vector group. (D, E) Expression levels of NDRG2 and GLT‐1 mRNA among different groups. *n* = 4. (F) Protein expression levels of NDRG2 and GLT‐1 in SDH. GAPDH was used as the internal control. (G, H) Relative protein expression levels of NDRG2 (G) and GLT‐1 (H) in each group. *n* = 4. (I) Changes in MWT among different groups of rats. (J) Changes in TWL among different groups of rats. *n* = 10. *p < 0.05, **p < 0.01 versus CCI + Vector group. ^#^ *p* < 0.05, ^##^ *p* < 0.01 versus CCI + Vector + Tuina group. Data are presented as mean ± SD.

To directly test the involvement of the NDRG2/GLT‐1 pathway in Tuina‐mediated analgesia, NDRG2 was overexpressed through intrathecal injection of rAAV‐GfaABC1D‐NDRG2‐P2A‐mCherry‐WPRE‐SV40 pA, administered 21 days before CCI surgery. Tuina treatment commenced on day 4 post‐CCI, with behavioural assessments conducted at multiple time points (Figure 4A). Fluorescence microscopy confirmed successful spinal cord infection (Figure 4B) and RT‐qPCR assay was performed to verify robust NDRG2 overexpression in the SDH with high infection efficiency (Figure 4C).

Subsequent RT‐qPCR and western blot analyses showed that AAV‐mediated NDRG2 overexpression significantly elevated astrocytic NDRG2 levels and suppressed GLT‐1 expression compared with the CCI + Vector group. Moreover, NDRG2 overexpression markedly attenuated the Tuina‐induced downregulation of NDRG2 and upregulation of GLT‐1 observed in the CCI + Vector+Tuina group (Figure 4D–H). These results confirm that the NDRG2/GLT‐1 pathway is critical for Tuina‐induced analgesia.

As expected, pain behaviour assessments showed that NDRG2 overexpression did not alter mechanical or thermal sensitivity in normal rats but significantly exacerbated pain responses in CCI rats. Furthermore, compared with the CCI + Vector + Tuina group, AAV‐NDRG2 markedly reduced the analgesic effect of Tuina (Figure 4I,J).

### 3.5 AAV‐NDRG2 Attenuates the Interaction Between Astrocytes and Synapses Induced by Tuina in CCI Rats

**FIGURE 5:**
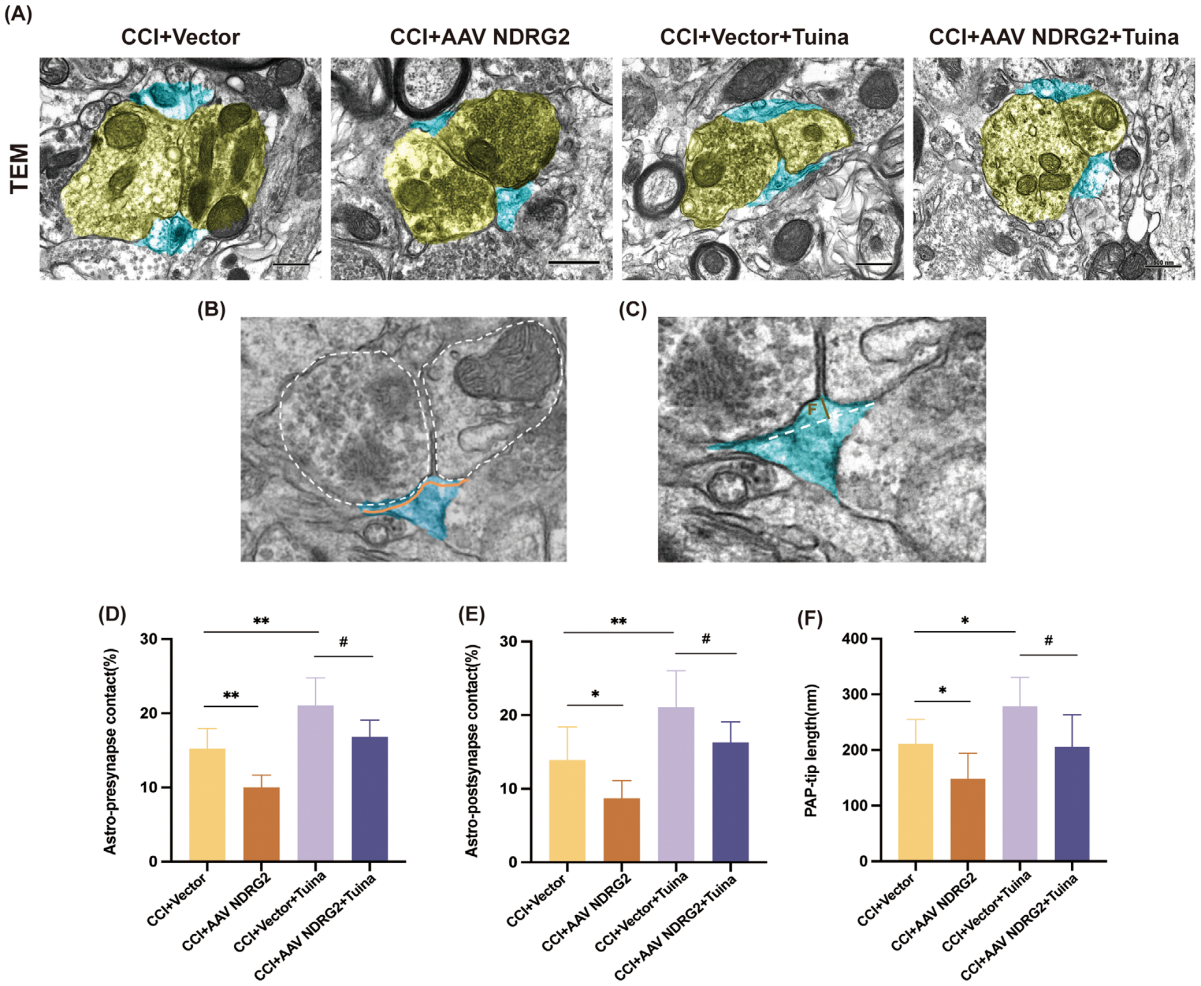
AAV‐NDRG2 attenuates the interaction between astrocytes and synapses induced by Tuina in CCI rats. (A) Extent of overlap between astrocytes and synapses in rat SDH of each group. Astrocytes and synapses are pseudo‐coloured in blue and yellow, respectively. Scale bar, 500 nm. (B) Example TEM image of a synapse in rat SDH. An outline of the pre‐synaptic and post‐synaptic membranes is indicated with a dashed line. Orange lines indicate the membrane interaction between pre‐synapse and post‐synapse with the astrocytes. (C) Example of measuring PAP tip length. (D) The astrocyte and pre‐synapse's percentage (%) of shared contact with respect to the pre‐synapse perimeter. (E) The astrocyte and post‐synapse's percentage (%) of shared contact with respect to the post‐synapse perimeter. The proportion of shared contact between the astrocyte and post‐synapse, as measured by the post‐synapse perimeter. (F) Astrocytic PAP tip length measurement. *n* = 3. Data are presented as mean ± SD. *p < 0.05, **p < 0.01 versus CCI + Vector group. ^#^ *p* < 0.05. versus CCI + Vector+Tuina group.

We next examined the role of AAV‐NDRG2 in modulating astrocyte–synapse interactions. TEM analysis revealed that, relative to the CCI + Vector group, intrathecal AAV‐NDRG2 injection significantly decreased the astrocyte coverage of synapses, whereas Tuina treatment enhanced this coverage. Importantly, compared with the CCI + Vector + Tuina group, AAV‐NDRG2 attenuated the Tuina‐induced increase in astrocyte–synapse association within the SDH of CCI rat models (Figure 5A).

To further characterise astrocyte–synapse interactions, we quantified the contact length between astrocytes and pre‐ or post‐synaptic sites, normalised to the circumference of each site. This metric defined the astrocyte–synapse interface. The schematic in Figure 5B illustrates how astrocytic processes constrain synaptic glutamate spillover. Additionally, we evaluated astrocyte–cleft interactions by measuring the length of PAP tips around synapses, as shown in Figure 5C.

Our results demonstrated that, relative to the CCI + Vector group, AAV‐NDRG2 reduced astrocyte coverage at both pre‐ and post‐synaptic sites and shortened PAP tip length. Tuina treatment effectively reversed these changes. Moreover, when compared with the CCI + Vector + Tuina group, AAV‐NDRG2 diminished Tuina's ability to enhance astrocyte–synapse interactions in the SDH (Figure 5D–F).

### 3.6 AAV‐NDRG2 Weakens Tuina's Effect in Promoting Glutamate Uptake and Synaptic Plasticity in **CCI** Rats

**FIGURE 6:**
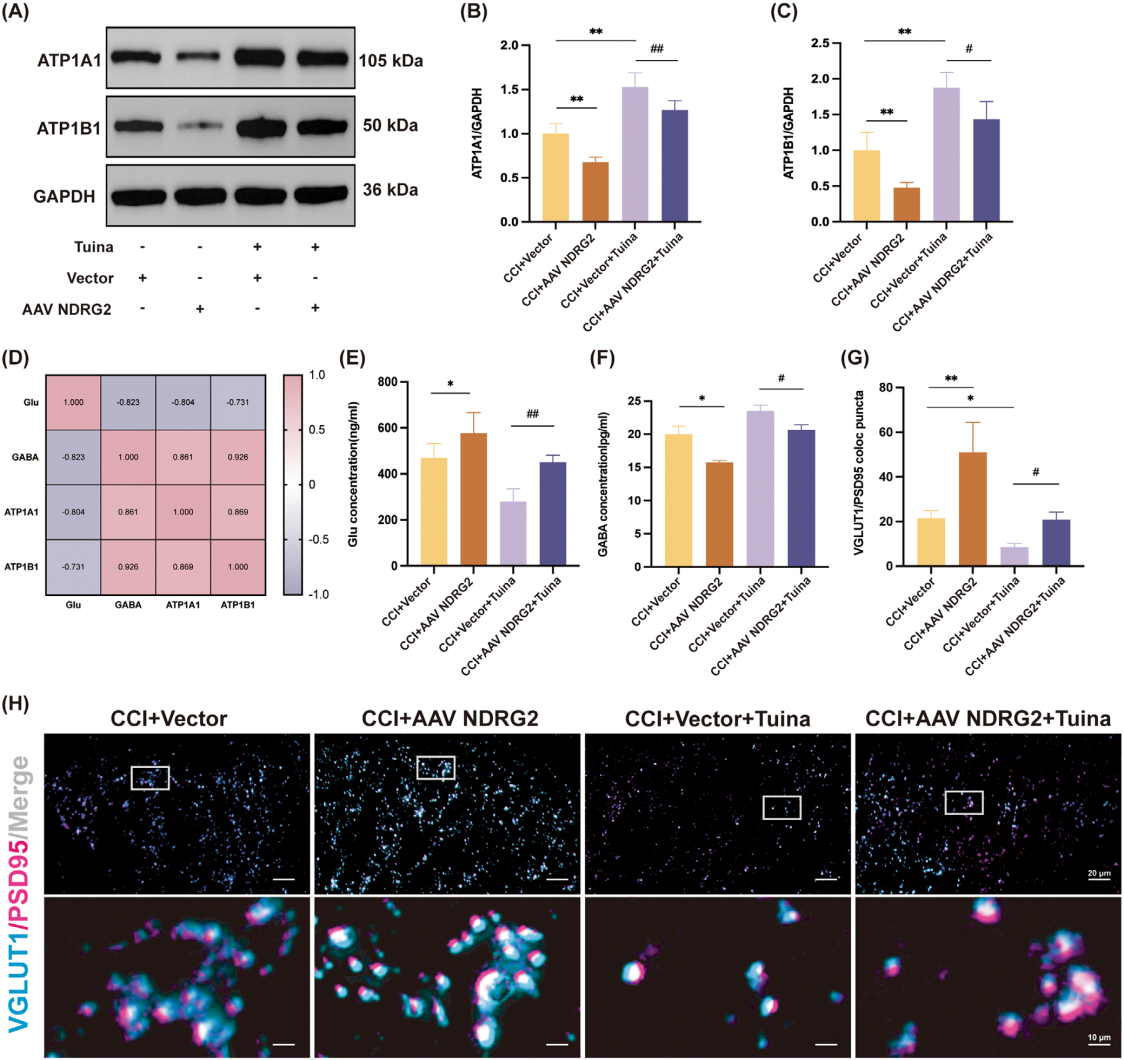
AAV‐NDRG2 weakens Tuina's effect in promoting glutamate uptake and synaptic plasticity in CCI rats. (A) Protein expression levels of ATP1A1 and ATP1B1 in SDH. GAPDH was used as the internal control. (B, C) Relative protein expression levels of ATP1A1 (B) and ATP1B1 (C) in each group. *n* = 4. (D) Pearson's correlation of glutamate and *γ*‐aminobutyric acid levels with ATP1A1 and ATP1B1 protein expression. (E, F) Concentrations of glutamate and *γ*‐aminobutyric acid in each group. *n* = 4. (G) Quantification analysis of the number of VGLUT1/PSD95 in SDH. *n* = 4. (H) Representative images of IF analysis of VGLUT1 (blue), PSD95 (pink) and Merge (white) in SDH among groups. Scale bar, 20 μm (top), 10 μm (bottom). Data are presented as mean ± SD *p < 0.05, **p < 0.01 versus CCI + Vector group. ^#^ *p* < 0.05, ^##^ *p* < 0.01 versus CCI + Vector+Tuina group.

To assess the impact of AAV‐NDRG2 on astrocytic glutamate transport and synaptic transmission efficiency, we measured the expression of Na^+^/K^+^‐ATPase subunits ATP1A1 and ATP1B1 (Figure 6A–C) and quantified glutamate and GABA concentrations (Figure 6E,F). Since efficient glutamate clearance relies on Na^+^/K^+^‐ATPase activity, these parameters serve as functional readouts. Compared with the CCI + Vector group, AAV‐NDRG2 reduced ATP1A1 and ATP1B1 expression, elevated glutamate concentration, and decreased GABA concentration. Furthermore, relative to the CCI + Vector + Tuina group, AAV‐NDRG2 blunted Tuina's upregulation of ATP1A1 and ATP1B1, thereby promoting glutamate accumulation.

Pearson correlation analysis revealed a negative correlation between glutamate concentration and ATP1A1/ATP1B1 expression, and a positive correlation between GABA concentration and these subunits (Figure 6D). These findings suggest that Tuina enhances glutamate clearance by increasing ATP1A1 and ATP1B1 expression, whereas NDRG2 overexpression counteracts this effect.

Collectively, these results demonstrate that Tuina promotes astrocytic ensheathment of synapses by suppressing NDRG2 expression, thereby improving synaptic function through efficient clearance of glutamate from the synaptic cleft. To further investigate synaptic regulation, we examined the co‐localization of the presynaptic marker VGLUT1 and the postsynaptic marker PSD95, which reflects excitatory synaptic transmission. Compared with the CCI + Vector group, NDRG2 overexpression significantly increased the number of VGLUT1/PSD95 double‐positive cells, whereas Tuina treatment reduced them. However, relative to the CCI + Vector + Tuina group, NDRG2 overexpression weakened Tuina's inhibitory effect (Figure 6G,H). These results indicate that NDRG2 overexpression diminishes Tuina's ability to suppress excitatory synaptic transmission.

## 4 Discussion

NP is a debilitating chronic condition with complex pathophysiology [17]. Tuina is grounded in a long history of application and a robust theoretical framework within traditional Chinese medicine for analgesia. Unlike conventional physical therapies that primarily modulate peripheral tissue or neurophysiological function, for example, via thermotherapy or neuromodulation, Tuina is fundamentally rooted in the meridian theory. It employs manipulation methods, such as kneading, pressing, and rolling, to stimulate superficial acupoints and meridians. This stimulation aims to warm and unblock meridians, promote the flow of qi and blood, and ultimately alleviate pain. In China, Tuina is extensively employed in the clinical management of pain. A meta‐analysis showed that Tuina was more effective than traction in managing NP associated with lumbar disc herniation [18]. Accumulating evidence substantiates the superiority of Tuina over physical therapies in ameliorating symptoms, enhancing physical function, and improving treatment satisfaction [19]. Furthermore, Tuina may serve as an alternative or adjunct to certain analgesics, potentially reducing reliance on pharmacotherapy and minimizing the associated risk of adverse events [20]. Consequently, given its clinically meaningful benefits, elucidating the molecular mechanisms underlying Tuina induced analgesia has become a major focus of scientific inquiry.

Through the application of mechanical force, Tuina activates peripheral receptors on the body surface, which then relay signals to the SDH via primary sensory neurons [21]. Evidence suggests that the analgesic effects of Tuina are mediated by alterations in excitatory neurotransmitters, inhibitory substances, inflammatory factors, and ion channels within the serum or dorsal root ganglion (DRG) [22]. Our prior research demonstrated that Tuina mitigates radicular pain and hyperalgesia in a CCI rat by diminishing serum SP levels, lowering DRG IL‐23 expression, and suppressing P2X3 receptor‐mediated currents in the DRG [23]. However, pain perception results from the central integration and modulation of peripheral inputs; therefore, a sole peripheral‐level focus is inadequate for a comprehensive mechanistic understanding. Therefore, extending our peripheral research, we investigated the primary central nervous system and identified the modulation of synaptic plasticity in the SDH as a mechanism underlying Tuina's therapeutic effect on NP.

In this study, Tuina significantly reduced mechanical and thermal hypersensitivity in CCI rats. These behavioural improvements were accompanied by suppressed astrocyte activation, reduced glutamate accumulation and favourable remodelling of astrocytic and synaptic structures. Specifically, Tuina decreased GFAP expression, lowered extracellular glutamate levels and downregulated the synaptic plasticity proteins Syp and Syt‐1.

Mechanistically, Tuina inhibited aberrant NDRG2 signalling by downregulating NDRG2 and restoring GLT‐1 expression in SDH astrocytes of CCI rats. Overexpression of NDRG2/GLT‐1 signalling pathway through AAV‐NDRG2 impaired glutamate clearance from the synaptic cleft, markedly attenuating Tuina's ability to normalise SDH plasticity. These findings indicate that Tuina, particularly through kneading at the Weizhong (BL40) acupoint, mitigates NP by suppressing pathological synaptic plasticity through the astrocytic NDRG2/GLT‐1 signalling pathway.

Consistent with prior studies, Tuina applied at the Weizhong acupoint produced significant analgesic effects in NP model rats [24, 25, 26]. We dynamically assessed MWT and TWL across multiple time points. As expected, MWT and TWL significantly declined 1 day after CCI surgery, replicating classical NP features [27]. From days 7 to 14 of Tuina intervention, both thresholds increased significantly compared to untreated CCI rats, confirming a cumulative analgesic effect. These results align with our previous findings [28, 29, 30]. Moreover, the analgesic profile of Tuina closely paralleled that of FCA injection, in terms of both onset and magnitude, providing preliminary evidence that Tuina achieves sustained pain relief through astrocyte‐mediated modulation of synaptic plasticity.

Astrocytes remain persistently activated following harmful stimuli or nerve injury, thereby contributing to the chronic course of NP [31, 32]. Our findings demonstrate that Tuina attenuated pathological morphological changes and structural damage in astrocytes, significantly reducing GFAP expression and reversing astrocytic activation, consistent with the findings of prior reports [33, 34]. Increasing evidence highlights the central role of astrocytes in regulating synaptic plasticity during NP progression [35, 36, 37]. In line with this, we observed that Tuina downregulated synaptic plasticity markers Syp and Syt‐1, potentially reflecting astrocyte‐mediated regulation of neurotransmitter homeostasis [38, 39, 40].

Glutamate, the primary excitatory neurotransmitter in nociceptive pathways [41], is pivotal in pain transmission by amplifying excitatory signalling in the spinal cord [42]. This effect is normally counterbalanced by the inhibitory transmitter GABA. When excitation–inhibition balance within the synaptic cleft is disrupted, excitatory drive intensifies, synaptic strength increases and NP ensues [43]. Astrocytes mitigate this imbalance by rapidly clearing excess glutamate through the high‐affinity transporter GLT‐1, regulated by NDRG2 and by converting intracellular glutamate into GABA via enzymatic pathways, thereby restoring homeostasis [44]. Consistent with this mechanism, we observed the high expression of NDRG2 and GLT‐1 in astrocytes. Tuina intervention reduced NDRG2 and enhanced GLT‐1 expression, effectively normalising glutamate and GABA levels in the SDH and correcting CCI‐induced glutamate dysmetabolism. These results suggest that the astrocytic NDRG2/GLT‐1 pathway is a key contributor to the analgesic effects of Tuina.

To clarify this mechanism, we overexpressed astrocytic NDRG2 via intrathecal injection of AAV‐NDRG2. NDRG2 promotes glutamate uptake by enhancing Na^+^/K^+^‐ATPase activity [45]. During GLT‐1‐mediated uptake, Na^+^/K^+^‐ATPase actively exchanges three Na^+^ for two K^+^, establishing an electrochemical gradient that reduces the energetic cost of glutamate transport against steep synaptic gradients [46, 47]. Mechanistically, NDRG2 stabilises the *β*1 subunit of Na^+^/K^+^‐ATPase by inhibiting its ubiquitination, facilitating *α*‐subunit trafficking to the plasma membrane and thereby enhancing catalytic efficiency and GLT‐1 function [48]. In line with prior work [49], we found an inverse correlation between glutamate concentration and the expression of Na^+^/K^+^‐ATPase subunits ATP1A1 and ATP1B1. Notably, NDRG2 overexpression abrogated Tuina‐induced upregulation of ATP1A1, ATP1B1 and GLT‐1 in CCI rats, thereby weakening glutamate clearance. Behaviorally, AAV‐NDRG2 injection exacerbated mechanical and thermal hypersensitivity, reducing Tuina's analgesic efficacy. These results indicate that Tuina alleviates NP primarily through the inhibition of astrocytic NDRG2 signalling.

Astrocytes sense and adhere to synapses during the fine‐tuning of glutamate regulation, thereby forming functional associations with them [50]. Considerable progress has been made in elucidating their role in regulating synaptic plasticity within NP research [51, 52]. Astrocytes exhibit morphological plasticity in response to synaptic activity and the perimeter of astrocyte–synapse contact serves as a key indicator of their spatial interaction [53, 54]. A longer contact interface corresponds to a stronger capacity to restrict the spillover of synaptic glutamate and other neuroactive molecules [55]. In this study, TEM analysis provided detailed morphological characterisation and quantitative assessment of astrocyte–synapse interactions. We found that NDRG2 overexpression reduced astrocytic coverage of both pre‐ and post‐synaptic regions, confirming that astrocyte–synapse interactions occur on both sides. Previous 3D modelling studies in the hippocampal CA1 region demonstrated that astrocytic processes are nearly threefold more abundant in postsynaptic than in presynaptic domains [56]. By contrast, our perimeter ratio analysis in the SDH revealed a more balanced astrocytic distribution across pre‐ and post‐synaptic sites. This discrepancy likely reflects regional variability in astrocyte morphology and synaptic coverage. Importantly, NDRG2 overexpression reversed the Tuina‐induced enhancement of astrocyte–synapse coverage in CCI rats, thereby attenuating Tuina's ability to limit glutamate spillover.

Furthermore, the efficiency of GLT‐1 transport is greatest in PAPs that penetrate deeply into the synaptic cleft (> 150 nm) [57]. We found that NDRG2 overexpression shortened PAP tip length, impairing this high‐efficiency uptake. Tuina promoted PAP elongation in CCI rats, but this effect was suppressed by NDRG2 overexpression. Thus, Tuina likely enhances glutamate clearance by inhibiting NDRG2, allowing deeper astrocytic invasion of the synaptic cleft and strengthening GLT‐1 activity.

Excitatory synaptic density, commonly quantified by VGLUT1–PSD95 co‐localization, serves as a reliable index of excitatory transmission [58, 59, 60]. Our results demonstrated that NDRG2 overexpression diminished Tuina's capacity to reduce excitatory glutamatergic synapse density, further supporting a mechanism in which Tuina mitigates excitatory transmission through astrocytic NDRG2 inhibition.

In summary, Tuina alleviates NP by suppressing astrocytic NDRG2 expression, thereby enhancing GLT‐1‐mediated glutamate uptake, promoting astrocyte–synapse interactions, extending PAPs into the synaptic cleft, reducing glutamate spillover and ultimately dampening excitatory synaptic plasticity.

However, several limitations should be considered. First, while our study used VGLUT1/PSD95 co‐localization as a structural correlate of synaptic strength, a method strongly associated with functional synaptic changes, direct electrophysiological recordings from spinal cord slices would provide valuable functional validation of the observed synaptic regulation. Second, the overexpression of NDRG2 only partially attenuated Tuina's analgesic effect, suggesting that, beyond the NDRG2/GLT‐1 signalling pathway, additional mechanisms likely contribute to Tuina's efficacy in alleviating neuropathic pain in CCI rats. To deepen the understanding of Tuina's analgesic mechanisms, future research should first employ electrophysiological methods to functionally confirm the synaptic regulation observed here. Subsequently, studies are needed to elucidate the additional pathways involved. We also plan to explore the potential roles of the central nervous system in mediating its multi‐level effects and to integrate optogenetics with in vivo calcium imaging to dynamically characterise the remodelling of pain‐related neural circuits.

## 5 Conclusion

**FIGURE 7:**
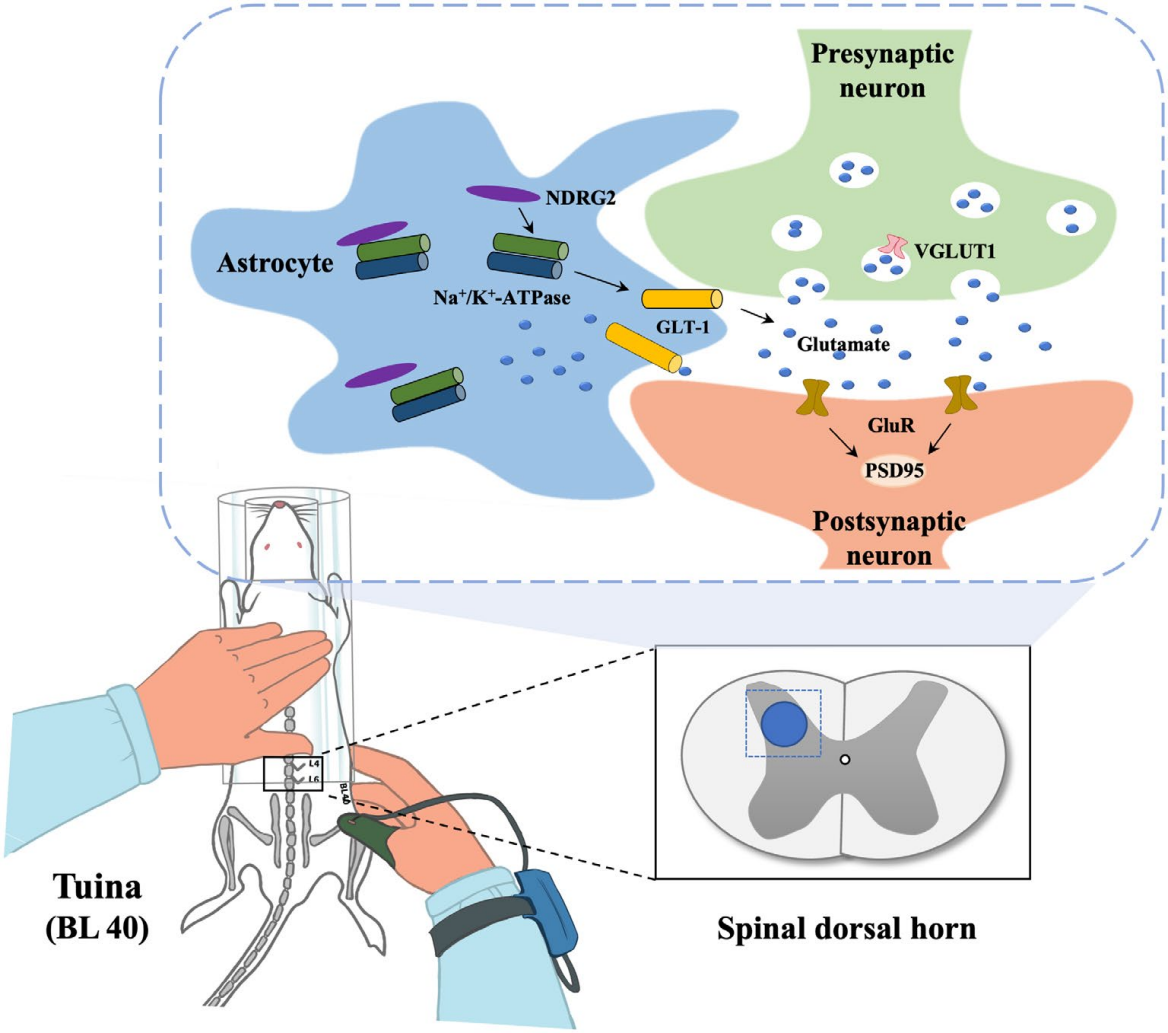
Schematic diagram showing the mechanism of Tuina manipulation in treating neuropathic pain.

This study demonstrates that Tuina intervention significantly alleviates CCI‐induced pain hypersensitivity and effectively reverses astrocyte‐mediated synaptic plasticity. Our findings suggest that Tuina suppresses astrocytic NDRG2 expression and enhances GLT‐1 activity, thereby promoting efficient glutamate clearance. Notably, NDRG2 overexpression diminished Tuina's effects on synaptic plasticity (Figure 7). Collectively, these results indicate that the astrocytic NDRG2/GLT‐1 signalling pathway, which regulates synaptic plasticity in the SDH, may represent a novel therapeutic target of Tuina in NP treatment. Continued research on this pathway will not only improve our understanding of Tuina's analgesic mechanism but also support its translation into clinical practice.

## Author Contributions

**Huanzhen Zhang:** data curation (lead); methodology (equal); writing – original draft (lead); funding acquisition (lead). **Lechun Chen:** investigation (lead); methodology (equal). **Jingjing Jiang:** investigation (equal); methodology (equal). **Limei Huang:** writing – review and editing (equal); funding acquisition (lead). **Hongye Huang:** software (equal); visualization (equal). **Lanting Huang:** data curation (equal); formal analysis (equal). **Shuijin Chen:** conceptualization (lead); supervision (lead). **Zhigang Lin:** writing – review and editing (lead); funding acquisition (lead).

## Funding

This study was supported by National Natural Science Foundation of China, 82105039, 82575244, 82505785. Natural Science Foundation of Fujian Province, 2022J01881, 2024J01141.

## Conflicts of Interest

The authors declare no conflicts of interest.

## Data Availability

The data that support the findings of this study are available from the corresponding author upon reasonable request.
